# New physics searches at the BESIII experiment

**DOI:** 10.1093/nsr/nwab189

**Published:** 2021-10-20

**Authors:** Shenjian Chen, Stephen Lars Olsen

**Affiliations:** School of Physics, Nanjing University, Nanjing 210093, China; Nanjing Proton Source Research and Design Center, Nanjing 210093, China; University of Chinese Academy of Sciences, Beijing 100049, China; Institute for Basic Science, Daejeon 34126, South Korea

**Keywords:** new physics, dark photons, lepton flavor violation, *C* and *CP* violation

## Abstract

The standard model (SM) of particle physics, comprised of the unified electroweak and quantum chromodynamic theories, accurately explains almost all experimental results related to the micro-world, and has made a number of predictions for previously unseen particles, most notably the Higgs scalar boson, that were subsequently discovered. As a result, the SM is currently universally accepted as the theory of the fundamental particles and their interactions. However, in spite of its numerous successes, the SM has a number of apparent shortcomings, including: many free parameters that must be supplied by experimental measurements; no mechanism to produce the dominance of matter over antimatter in the universe; and no explanations for gravity, the dark matter in the universe, neutrino masses, the number of particle generations, etc. Because of these shortcomings, there is considerable incentive to search for evidence for new, non-SM physics phenomena that might provide important clues about what a new, beyond the SM theory (BSM) might look like. Although the center-of-mass energies that BESIII can access are far below the energy frontier, searches for new, BSM physics are an important component of its research program. This report reviews some of the highlights from BESIII’s searches for signs of new, BSM physics by: measuring rates for processes that the SM predicts to be forbidden or very rare; searching for non-SM particles such as dark photons; performing precision tests of SM predictions; and looking for violations of the discrete symmetries *C* and *CP* in processes for which the SM expectations are immeasurably small.

## Teaser

This report reviews some highlights of the BESIII’s activities in searching for new physical signs that deviate from the predictions of the Standard Model of particle physics.

## INTRODUCTION

The standard model consistently predicts the results of experimental measurements and has emerged as the only viable candidate theory for describing elementary particle interactions [1–4]. In spite of its great success, there are a number of reasons to believe that the standard model (SM) is not the ultimate theory, including the following.

- The SM has 19 free parameters that must be supplied by experimental measurements. These include the quark, lepton and Higgs masses, the mixing angles of the Cabibbo–Kobayashi–Maskawa (CKM) quark-flavor mixing matrix, and the couplings of the electric, weak and quantum chromodynamic (QCD) color forces.
- As first pointed out by Sakharov [5], the matter-antimatter asymmetry of the universe implies the existence of sizable *CP*-violating interactions in nature. However, the established SM mechanism for *CP* violation fails to explain the matter-dominated universe by about 10 orders of magnitude; there must be additional *CP*-violating mechanisms in nature beyond those contained in the SM.
- The model has no explanation for dark matter, which is, apparently, the dominant component of the mass of the universe.
- The particles in the SM are arranged in three generations of colored quarks and three generations of leptons; particle interactions are mediated by three forces: the color, electromagnetic and weak forces. The theory provides no explanation for why the number of generations is three and it does not account in any way for gravity, the fourth force that is known to exist.

As a result, there have been a huge number of experimental efforts aimed at finding ‘new physics,’ which refers to new physical phenomena beyond the standard model (BSM) of particle physics. This may be, for example, a new fundamental particle, such as a fourth generation quark or lepton, or a new fundamental force carrier, such as a dark photon, high-mass gauge boson, a new Higgs-like meson, etc. Searches for new physics can be performed in two ways. One method is to look for direct production of new particles in collisions at high-energy accelerators, for example at the Large Hadron Collider, and reconstruct it from its SM decay products. Another way is to measure precisely a decay process that can be accurately described by the SM, and look for deviations from the SM prediction of the decay rate. According to quantum field theory (QFT), new heavy particles can contribute to the decay process through virtual loop diagrams. These make precision measurements sensitive to new physics, and this technique is widely used in high intensity collider experiments such as BESIII [6–8].

Here we review highlights of some of these activities at BESIII.

## RARE PROCESSES

### Search for flavor changing neutral currents

Flavor changing neutral current (FCNC) processes transform an up-type (*u*, *c*, *t*) or down-type (*d*, *s*, *b*) quark into another quark of the same type but with a different flavor. In the SM, these processes are mediated by the *Z* boson and are known as neutral currents. However, they are strongly suppressed by the Glashow-Iliopoulos-Maiani (GIM) cancelation [9] and only occur as second-order loop processes. In many extensions of the SM, virtual TeV-scale particles can contribute competing processes that lead to measurable deviations from SM-inferred transition rates or other properties. Hence, studies of rare FCNC processes are suitable probes for new physics.

Recently, hints of discrepancies have been observed in the semi-leptonic FCNC processes of the *b* quark, *b* → *s*ℓ^+^ℓ^−^ (ℓ = *e*, μ), by the LHCb experiment [10]. (1) The differential branching fractions measured as a function of the squared four-momentum transferred to the two leptons, *Q*^2^, for several *B*-meson decay modes are below the theoretical predictions [11–15]. The largest local discrepancy is a 3.3σ difference in the rate for $B_s^0\rightarrow \phi \mu ^+\mu ^-$ decay from its SM-predicted value. (2) The ratios of branching fractions for decays involving muons and electrons, defined as

$$\begin{eqnarray*}
R_K&=& \frac{\mathcal {B}(B^+\rightarrow K^+\mu ^+\mu ^-)}{\mathcal {B}(B^+\rightarrow K^+e^+e^-)} \\
\text{and}\quad R_{K^*}&=& \frac{\mathcal {B}(B^+\rightarrow K^{*+}\mu ^+\mu ^-)}{\mathcal {B}(B^+\rightarrow K^{*+}e^+e^-)},
\end{eqnarray*}$$

which are unity in the SM (i.e. lepton-flavor universality), were measured to be [16,17]

$$\begin{eqnarray*}
R_K&=& 0.745^{+0.090}_{-0.074}\pm 0.036 \text{ at central }\\
&&Q^2\in [1.0, 6.0]\, \text{GeV}/c^2, \quad 2.6\sigma , \\
R_{K^*} &=& 0.66^{+0.11}_{-0.07}\pm 0.03 \text{ at low }\\
&&Q^2\in [0.045, 1.1] \text{GeV}/c^2, \quad\!\!\! 2.1\sigma -2.3\sigma , \\
R_{K^*} &=& 0.69^{+0.11}_{-0.07}\pm 0.05 \text{ at central }\\
&&Q^2\in [1.1, 6.0]\, \text{GeV}/c^2, \quad 2.4\sigma -2.5\sigma ,
\end{eqnarray*}$$

where the levels of deviations from the SM predictions are indicated. (3) Measurements of the quantity $P^{\prime }_5$, which is the chiral asymmetry produced by the interference between the transversely and longitudinally polarized amplitudes in the decay *B* → K^* +^ℓ^+^ℓ^−^, are 2.8σ and 3.0σ lower than the SM prediction in two *Q*^2^ intervals below the *J*/ψ resonance mass [18]. Since these discrepancies could be evidence for new particles that would extend the SM, it is important to check if there are similar deviations in the charm sector.

While SM rates for FCNC transitions in the down-type *b*- or *s*-quark sectors are relatively frequent because of the large mass of the top quark contribution to the loop, those in the up-type *c*-quark sector are especially rare due to the small masses of the intermediate down-like quarks in the loop that result in a strong GIM cancelation. For *c* → *u* transition rates for charmed and charmonia particles that proceed via the SM loop contribution, dubbed short distance effects, the expected branching fractions are typically between <10^−8^ [19–24] and 10^−10^–10^−14^ [25–27], respectively. For FCNC decays of charmed mesons, the measured rates are enhanced by a few orders of magnitude by SM contributions from long distance effects that proceed via di-lepton decays of ordinary ρ, ω and φ vector mesons [23,24]. However, some extensions to the SM further enhance these FCNC processes, sometimes by orders of magnitude [22,28–32].

The BESIII experiment has searched for *c*-quark FCNC processes in both charmed meson and charmonium decays. No significant signals for new physics are found in any of the investigated decay modes, and the inferred 90% confidence level (CL) upper limits on the branching fractions are summarized in Table 1.

- For the *D*^0^ → γγ mode, the upper limit is consistent with that previously set by the BaBar experiment [33]. The BESIII result is the first experimental study of this decay that uses *D*^0^ mesons produced at the open-charm threshold.
- For the rare decays $D\rightarrow h(h^{(^{\prime })}) e^+e^-$, where *h* indicates a meson that is comprised of *u*, *d*, and *s* quarks, searches for four-body decays of *D*^+^ mesons are performed for the first time, and the upper limits for *D*^0^ meson decays are, in general, one order of magnitude better than previous measurements [34].
- Searches for the FCNC decays ψ(2*S*) → *D*^0^*e*^+^*e*^−^ and $\psi (2S)\rightarrow \Lambda _c^+\bar{p} e^+e^-$ are performed for the first time. The upper limit on *J*/ψ → *D*^0^*e*^+^*e*^−^ is 2 orders of magnitude more stringent than the best previous result, which was set by the BESII collaboration [35].

**Table 1.** Results for the upper limit at the 90% CL on the branching fractions for various FCNC process searches performed at BESIII. Also listed are the best previous results and the SM predictions, where the branching fraction calculations for charmed meson and charmonium decays are based on long distance and short distance contributions, respectively.

		$\mathcal {B}^{\text UL}$ at		Previous		SM	
Mode	Data	90% CL	Ref.	best $\mathcal {B}^{\text UL}$	Ref.	prediction	Ref.
*D* ^0^ → γγ	2.92 fb^−1^ ψ(3770)	3.8 × 10^−6^	[36]	2.2 × 10^−6^	[33]	3.5 × 10^−8^	[20]
*D* ^+^ → π^+^π^0^*e*^+^*e*^−^	2.93 fb^−1^ ψ(3770)	1.4 × 10^−5^	[37]				
*D* ^+^ → *K*^+^π^0^*e*^+^*e*^−^	2.93 fb^−1^ ψ(3770)	1.5 × 10^−5^	[37]				
$D^+\rightarrow K_S^0\pi ^+ e^+e^-$	2.93 fb^−1^ ψ(3770)	2.6 × 10^−5^	[37]				
$D^+\rightarrow K_S^0\,\, K^+ e^+e^-$	2.93 fb^−1^ ψ(3770)	1.1 × 10^−5^	[37]				
*D* ^0^ → *K*^−^*K*^+^*e*^+^*e*^−^	2.93 fb^−1^ ψ(3770)	1.1 × 10^−5^	[37]	3.15 × 10^−4^	[34]	6.5 × 10^−7^	[24]
*D* ^0^ → π^+^π^−^*e*^+^*e*^−^	2.93 fb^−1^ ψ(3770)	0.7 × 10^−5^	[37]	3.73 × 10^−4^	[34]	2.0 × 10^−6^	[24]
*D* ^0^ → *K*^−^π^+^*e*^+^*e*^−^	2.93 fb^−1^ ψ(3770)	4.1 × 10^−5^	[37]	3.85 × 10^−4^	[34]	1.6 × 10^−5^	[24]
*D* ^0^ → π^0^*e*^+^*e*^−^	2.93 fb^−1^ ψ(3770)	0.4 × 10^−5^	[37]	0.45 × 10^−4^	[34]	0.8 × 10^−6^	[21]
*D* ^0^ → η*e*^+^*e*^−^	2.93 fb^−1^ ψ(3770)	0.3 × 10^−5^	[37]	$\phantom{0}1.1\times 10^{-4}$	[34]		
*D* ^0^ → ω*e*^+^*e*^−^	2.93 fb^−1^ ψ(3770)	0.6 × 10^−5^	[37]	$\phantom{0}1.8\times 10^{-4}$	[34]		
$D^0\rightarrow K_S^0 e^+e^-$	2.93 fb^−1^ ψ(3770)	1.2 × 10^−5^	[37]	$\phantom{0}1.1\times 10^{-4}$	[34]		
*J*/ψ → *D*^0^*e*^+^*e*^−^	1.31 B *J*/ψ	8.5 × 10^−8^	[38]	$\phantom{0}1.1\times 10^{-5}$	[35]	4.8 × 10^−14^	[27]
ψ(2*S*) → *D*^0^*e*^+^*e*^−^	448 M ψ(2*S*)	1.4 × 10^−7^	[38]				
$\psi (2S)\rightarrow \Lambda _c^+\bar{p}e^+e^-$	448 M ψ(2*S*)	1.7 × 10^−6^	[39]				

### Prospects for BESIII rare decay searches

The BESIII FCNC search results mentioned above are based on data collected in 2009–2012, which included 1.31B *J*/ψ and 448M ψ(2*S*) event samples and a 2.93 fb^−1^ data sample that was accumulated at *E*_CM_ = 3.773 MeV, the peak energy of the $\psi (3770)\rightarrow D\bar{D}$ resonance. BESIII has recently increased the *J*/ψ data sample to 10B events and will eventually increase the ψ(2*S*) sample to 3B events, and the $\psi (3770)\rightarrow D\bar{D}$ data to 20 fb^−1^ (see Table 7.1 of [40]). Since the results listed in Table 1 are mainly limited by statistics, when the full data are available and analyzed, the sensitivity levels of FCNC searches should improve, in most cases, by factors of ∼7, and decay branching fractions will be probed at the 10^−6^–10^−8^ levels. If no interesting signals are found, more stringent upper limits would be established that should further constrain the parameter spaces of a number of new physics models.

In contrast to FCNC processes, charged-current weak decays of charmonium states are allowed, but are expected to occur as very rare processes; the SM-predicted branching fractions are of the order 10^−10^–10^−8^ [25], which means that they would be difficult to detect at BESIII, even with the full 10B event *J*/ψ data sample. However, some BSM calculations based on a two-Higgs-doublet model predict that the branching ratios of charmonium weak decays could be enhanced to be as large as 10^−5^ [41]. BESIII searched for several Cabibbo-favored weak decays, such as the hadronic processes $J/\psi \rightarrow D_s^-\rho ^+$ and $J/\psi \rightarrow \bar{D}^0\bar{K}^{*0}$ [42], and the semi-leptonic process $J/\psi \rightarrow D_s^{(*)-}e^+\nu _e$ [43], and established 90% CL branching fraction upper limits in the ∼10^−5^–10^−6^ range. Searches for some Cabibbo-suppressed weak decays of the *J*/ψ are currently underway at BESIII, with expected branching fraction sensitivity levels of about 10^−7^.

## TESTING SM PREDICTIONS FOR LEPTON COUPLINGS AND CKM MATRIX ELEMENTS

In the SM, the strength of charged-current weak interactions is governed by a single universal parameter, the Fermi constant *G*_F_. The three charged leptons (*e*^−^, μ^−^, τ^−^) all couple to the *W* boson with this strength, a feature called lepton-flavor universality (LFU). Although the quarks appeared, at first, to have different coupling strengths, this is because of a misalignment between the charge =−1/3 strong-interaction flavor eigenstates (*d*, *s*, *b*) and their weak-interaction counterparts (*d*^′^, *s*^′^, *b*^′^), as was first realized by Cabibbo in 1963 [44]. He hypothesized that the weak interaction flavor states were related to the strong-interaction states by an orthogonal rotation; the most general rotation matrix for three quark generations was first written down by Kobayashi and Maskawa in 1973 [45]. The universality of the quark-*W* couplings is reflected by the unitarity of the CKM matrix. The equality of the weak interaction-coupling strengths for the quarks and leptons is a feature that is specific to the SM and is violated by many beyond-the-SM theories, such as those that include fourth generation quarks, additional weak vector bosons or multiple Higgs particles.

### Search for violations of charged lepton flavor universality

The equality of the electron and muon couplings, *g*_*e*_ and *g*_μ_, has been established at the ${\mathcal {O}}(0.2\%)$ level, i.e. (*g*_*e*_/*g*_μ_ − 1) = 0.002 ± 0.002, by a comparison between the *K*^+^ → *e*^+^ν_*e*_ and *K*^+^ → μ^+^ν_μ_ partial decay widths measured by the NA62 experiment [46] together with values from the Particle Data Group (PDG) for the *K*^+^ lifetime and the electron and muon masses [47]. The best test of the equality of the τ-lepton coupling and muon couplings, (*g*_τ_/*g*_μ_ − 1) = 0.0008 ± 0.0021, has similar precision and is from a BESIII measurement of the tau mass [48] together with PDG values of the tau-lepton’s lifetime and leptonic decay branching fractions.

The possibility of LFU violation has attracted considerable recent attention because of measurements from BaBar [49], Belle [50] and LHCb [51] of the relative decay rates for the semi-leptonic processes $\bar{B}\rightarrow D^{(*)}\tau ^-\nu$ and $\bar{B}\rightarrow D^{(*)}\ell ^-\nu$ (ℓ^−^ = μ^−^ or *e*^−^) that seem to violate SM expectations. Specifically, the Heavy Flavor Averaging Group’s recent averages of experimental measurements are [52]

(1) \begin{eqnarray*} {\mathcal {R}}_D &=& \frac{{\mathcal {B}}(\bar{B}\rightarrow D\tau ^-\nu )}{{\mathcal {B}}(\bar{B}\rightarrow D\ell ^-\nu )}\nonumber \\ &=& 0.340\pm 0.027 \pm\, 0.013 ({\rm expt.})\nonumber\\ &&\quad [\text{{\bf SM}:} 0.299\pm 0.003], \nonumber \\ {\mathcal {R}}_{D^*} &=& \frac{{\mathcal {B}}(\bar{B}\rightarrow D^{*}\tau ^-\nu )}{{\mathcal {B}}(\bar{B}\rightarrow D^{*}\ell ^-\nu )}\nonumber \\ &=&0.295\pm 0.011 \pm\, 0.008\,\, ({\rm expt.})\nonumber\\ &&\quad [\text{{\bf SM}:} 0.258\pm 0.005]. \end{eqnarray*}

Here the discrepancies with LFU, if they are real and not just statistical fluctuations, are of order 10%, and motivate more careful checks of LFU in semi-leptonic and purely leptonic charmed particle decays with BESIII data.

#### BESIII tests of LFU

Charmed particle decay measurements at BESIII are summarized in detail elsewhere in this journal volume [53]. Table 2 summarizes measurements that are relevant for LFU tests, where all the measurements agree with SM expectations within 1 ∼ 2σ. The quantities in the last column, $\sqrt{(\Gamma _{e(\tau )}/\Gamma _\mu )/{\rm SM}}-1$, which would be (*g*_*e*(τ)_/*g*_μ_ − 1) if radiative corrections and detailed considerations of the relevant form factors were properly applied, are included as indicators of the sensitivity levels. According to these values, the most stringent BESIII sensitivity levels for LFU-violating effects are a factor of 5 better than those of the $\bar{B}\rightarrow D^{(*)}\tau ^-\nu$ measurements (equation (1)) but an order of magnitude poorer than the limits on *g*_*e*_/*g*_μ_ from the *K*^+^ decay.

**Table 2.** BESIII measurements of charmed particle semi-leptonic and purely leptonic branching-fraction measurements, and comparisons of the Γ_*e*(τ)_/Γ_μ_ to SM expectations for LFU.

Mode	*n*_evts_	${\mathcal {B}}\ (\times 10^{-3})$	Ref.	Γ_*e*(τ)_/Γ_μ_	SM pred.	$\sqrt{\frac{\Gamma _{e(\tau )}/\Gamma _\mu }{\rm SM}}-1$
*D* ^0^ → *K*^−^μ^+^ν_μ_	47.1K	34.13 ± 0.19 ± 0.35	[54]	1.027 ± 0.014	1.026 ± 0.001	0.001 ± 0.008
*D* ^0^ → *K*^−^*e*^+^ν_*e*_	70.7K	35.05 ± 0.14 ± 0.33	[55]			
*D* ^0^ → π^−^μ^+^ν_μ_	2.3K	2.72 ± 0.08 ± 0.06	[56]	1.085 ± 0.037	1.015 ± 0.002	0.034 ± 0.019
*D* ^0^ → π^−^*e*^+^ν_*e*_	6.3K	2.95 ± 0.04 ± 0.03	[55]			
$D^+\rightarrow \bar{K}^0\mu ^+\nu _{\mu }$	20.7K	87.2 ± 0.7 ± 1.8	[57]	1.012 ± 0.033	≈1.03	
$D^+\rightarrow \bar{K}^0 e^+\nu _e$	26.0K	86.0 ± 0.6 ± 1.5	[58]			
*D* ^+^ → π^0^μ^+^ν_μ_	1.3K	3.50 ± 0.11 ± 0.10	[56]	1.037 ± 0.045	1.015 ± 0.002	0.011 ± 0.023
*D* ^+^ → π^0^*e*^+^ν_*e*_	3.4K	3.63 ± 0.08 ± 0.05	[58]			
*D* ^+^ → ωμ^+^ν_μ_	194	1.77 ± 0.18 ± 0.11	[59]	0.92 ± 0.14	0.93 − 0.97	
*D* ^+^ → ω*e*^+^ν_*e*_	491	1.63 ± 0.11 ± 0.08	[60]			
*D* ^+^ → ημ^+^ν_μ_	234	1.04 ± 0.10 ± 0.05	[61]	1.03 ± 0.13	1.0 − 1.03	
*D* ^+^ → η*e*^+^ν_*e*_	373	1.07 ± 0.08 ± 0.05	[62]			
$\Lambda _{c}^+\rightarrow \Lambda \mu ^+\nu _{\mu }$	79	34.9 ± 4.6 ± 2.7	[63]	1.04 ± 0.31	≈1.0	
$\Lambda _{c}^+\rightarrow \Lambda e^+\nu _e$	104	36.3 ± 3.8 ± 2.0	[64]			
*D* ^+^ → τ^+^ν_τ_	137	1.20 ± 0.24 ± 0.12	[65]	3.21 ± 0.77	2.67	0.096 ± 0.132
*D* ^+^ → μ^+^ν_μ_	409	0.37 ± 0.02 ± 0.01	[66]			
$D_{s}^+\rightarrow \tau ^+\nu _{\tau }$	4.9K	52.7 ± 1.0 ± 1.2	[67]	9.72 ± 0.37	9.75	−0.002 ± 0.019
$D_{s}^+\rightarrow \mu ^+\nu _{\mu }$	1.1K	5.49 ± 0.16 ± 0.15	[68]			

#### Future prospects for LFU tests at BESIII

The most stringent BESIII tests for LFU-violating effects in charmed-particle decays are derived from measurements of $D\rightarrow \bar{K}\ell ^+\nu$ and πℓ^+^ν semi-leptonic decays, where the current (*g*_*e*_/*g*_μ_ − 1) sensitivities are at the $1\%\sim 2\%$ level. These results are based on the analysis of the 2.97 fb^−1^ data sample accumulated at $\psi (3770)\rightarrow D\bar{D}$ resonance. When the analysis of the full 20 fb^−1^ data set is complete, the sensitivity levels of the LFU tests, which are now mostly statistically limited, will improve by factors of ∼2.5, and be in the sub-1% range. In this case, if the current 1.8σ discrepancy that BESIII sees in *D*^0^ → *K*^−^ℓ^−^ν is real and the central value reported in Table 2 persists, its significance will increase to more than 4σ. The other BESIII measurement with interesting potential is the ratio of the $D_{s}^+\rightarrow \tau ^+\nu$ and $D_{s}^+\rightarrow \mu ^+\nu$ purely leptonic decay rates that is based on analyses of a 3.19 fb^−1^ data sample collected at *E*_CM_ = 4178 MeV, where $\sigma (e^+e^-\rightarrow D_{s}^{*+}\bar{D}_s^-)$ has a local maximum of ∼1 nb. In this case, the BESIII long-range plan includes an additional 3 fb^−1^ data sample at 4178 MeV, which would provide a $\sqrt{2}$ improvement in (*g*_τ_/*g*_μ_ − 1) sensitivity.

### Unitarity of the CKM matrix and the Cabibbo angle anomaly

**Figure 1.**
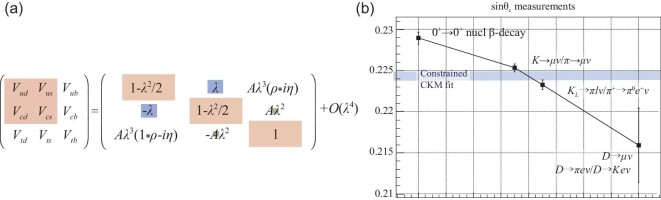
(a) The CKM matrix and its Wolfenstein parameterization. The shaded rectangles in the latter have areas ∝|*V*_*ij*_|. (b) Values of sin θ_*C*_ derived from different measurements. The value based on nuclear β decay is from [70], the one from *K*_μ2_ (*K*_ℓ3_) decays is from [72] ([75]), and the one from *D* decays is the average of BESIII ${\mathcal {B}}(D^+\rightarrow \mu ^+\nu )$ [66] and ${\mathcal {B}}(D^0\rightarrow \pi ^-e^+\nu )/{\mathcal {B}}(D^0\rightarrow K^- e^+\nu )$ [55] measurements. The shaded blue band is the PDG 2018 sin θ_*C*_ value based on a unitarity-constrained fit to all CKM elements [47].

The CKM matrix (see Fig. 1(a)) is the DNA of flavor physics; its elements characterize all of the SM weak charged current interactions of quarks. It defines a rotation in three dimensions of flavor space and, in the SM where there are three quark generations, it must be exactly unitary; any deviation from this would be a clear signal for new physics.

The unitarity condition for the top row of the CKM matrix is: |*V*_*ud*_|^2^ + |*V*_*us*_|^2^ + |*V*_*ub*_|^2^ = 1. Experimentally, a high-precision value of |*V*_*ud*_| comes from an analysis of eight superallowed 0^+^ → 0^+^ nuclear β decays [69] corrected for electroweak effects. The latest result is |*V*_*ud*_| = 0.97370(4) [70]. A precise value of the ratio |*V*_*us*_|/|*V*_*ud*_| = 0.2313(5) is determined from a KLOE measurement of ${\mathcal {B}}(K^+\rightarrow \mu ^+\nu )$ [71], the PDG 2018 world average for ${\mathcal {B}}(\pi ^+\rightarrow \mu ^+\nu )$ [47] and a Flavour Lattice Averaging Group average of LQCD evaluations of the pseudoscalar form-factor ratio $f_{K^+}/f_{\pi ^+}$ [72]. The value of |*V*_*ub*_|^2^, determined from *B*-meson decays, is $\sim {\mathcal {O}}(10^{-5})$ and is a negligible contributor to the unitarity condition [47]. The combination of these results [70],

(2) \begin{equation*} |V_{ud}|^2 +|V_{us}|^2 +|V_{ub}|^2 = 0.9983(5), \end{equation*}

indicates a nominal ∼3.5σ deviation from unitarity that, if taken at face value, is strong evidence for a SM violation.

Since deviations from CKM unitarity would be a clear sign of new physics, the equation (2) result inspired further investigation. These included: independent determinations of |*V*_*ud*_| based on the neutron lifetime [73,74] that returned consistent results, albeit with a slightly larger error; an independent evaluation of |*V*_*us*_|/|*V*_*ud*_| using ${\mathcal {B}}(K_L\rightarrow \pi \ell \nu )$ and ${\mathcal {B}}(\pi ^+\rightarrow \pi ^0e^+\nu )$ [75] that found an even larger deviation from unitarity, but with a correspondingly larger error; and re-examinations of the nuclear physics corrections used in the nuclear β-decay analyses for |*V*_*ud*_| [76,77] that did not change the central value, but indicated that the previous error that was assigned to these effects may have been somewhat underestimated. The current state of affairs is that the best current analyses of the existing data find an ${\mathcal {O}}(0.1\%)$ deviation from unitarity for the top row of the CKM matrix with a significance level that is somewhere in the 2σ ∼ 5σ range.

The strong generational hierarchy of the CKM quark-flavor mixing matrix is illustrated in Fig. 1(a), where the Wolfenstein parameterization [78] is shown with shaded rectangles with areas that are proportional to |*V*_*i*, *j*_|. Transitions between different generations (i.e. further off-diagonal elements) are successively suppressed by additional factors of λ = sin θ_*C*_ ≃ 0.225, where θ_*C*_ is the Cabibbo angle. A striking feature of the Wolfenstein formulation, and a characteristic of the SM, is that, to ${\mathcal {O}}(\lambda ^6)\sim 10^{-4}$, the four entries in the upper-left corner of the matrix, i.e. all transitions involving (*u*, *d*) and (*c*, *s*) quarks, are well characterized by the single parameter, sin θ_*C*_. Grossman *et al.* [79] argued that comparing the sin θ_*C*_ values derived from different *q*_*i*_ ↔ *q*_*j*_ (*i* = *u*, *c*;  *j* = *d*, *s*) subprocesses is a more sensitive test for new physics than tests of the CKM matrix unitarity, and provided, in support of this claim, an example of a toy model that has a heavy gauge boson with different *d*- and *s*-quark couplings that demonstrates this. In Fig. 1(b), values of sin θ_*C*_ derived from the nuclear β decay (*u* ↔ *d*) and *K*_ℓ2_ and *K*_ℓ3_ decay (*u* ↔ *s*) transitions discussed in the previous paragraph are shown. The apparent discrepancy from a single, universal value is referred to as the *Cabibbo angle anomaly*.

Studies of *c* → *d* transitions provide independent sin θ_*C*_ determinations. In the SM, |*V*_*cd*_| = |*V*_*us*_| = sin θ_*C*_; a deviation between the sin θ_*C*_ value inferred from *c* → *d* decays and that evaluated from *K*_ℓ2_ and *K*_ℓ3_ decays would be another clear indication of new physics. To date, this relation has not been strenuously tested. The PDG 2018 world-average value, |*V*_*us*_| = 0.2243 ± 0.0005, differs from that for |*V*_*cd*_| = 0.218 ± 0.004 by 1.5σ, with an uncertainty that is nearly an order of magnitude poorer [47]. The best determinations of |*V*_*cd*_| to date are from statistically limited BESIII measurements of ${\mathcal {B}}(D^+\rightarrow \mu ^+\nu )$ [66] and the ratio ${\mathcal {B}}(D^0\rightarrow \pi ^-e^+\nu )/{\mathcal {B}}(D^0\rightarrow K^- e^+\nu )$ [55], both of which are based on analyses of BESIII’s 2.97 fb^−1^ sample of $\psi (3770)\rightarrow D\bar{D}$ events that are discussed elsewhere in this journal volume [53]. The average value of the two |*V*_*cd*_| measurements is plotted in Fig. 1(b).

With the full 20 fb^−1^ ψ(3770) data sample, the BESIII precision on |*V*_*cd*_| should be improved by at least a factor of 2.5; if the result is the same as the current central value, the significance of the discrepancy would increase to about the 4σ level.

## SEARCHES FOR NON-SM SOURCES OF *CP* VIOLATION

Searches for new sources of *CP* violation have been elevated to a new level of interest by the recent LHCb discovery of a *CP*-violating asymmetry in the charmed quark sector; a 5σ difference between the branching fractions for *D*^0^ → *K*^+^*K*^−^ or π^+^π^−^ and $\bar{D}^0$ to the same final states, with a magnitude of order 10^−3^ [80]. The measured *CP*-violating asymmetry is at the high end of theoretical estimates for its SM value, which range from 10^−3^ [81–84] to 10^−4^ [85]. Although the LHCb result is intriguing in that it may be a sign of the long-sought-for non-SM mechanism for *CP* violation, uncertainties in the SM calculations for this asymmetry make it impossible to either establish or rule out this possibility [86].

Violations of *CP* have never been observed in weak decays of strange hyperons; the current limit on *CP*-violating asymmetry in Λ hyperon decay is of order 10^−2^ [87], which is 2 orders of magnitude above the highest conceivable SM effects [88]. A non-zero measurement of a *CP*-violating asymmetry at the level of ∼10^−3^ would be an unambiguous signature for new physics.

### Search for *CP* violation in Λ → *p*π^−^ decay

Parity violation in the weak interactions was discovered in 1957 [89,90]. Immediately thereafter there was considerable interest is studying parity violations in strange hyperon decays that were predicted by Lee and Yang [91]. For the *Y* → *B*π weak decay process, where *Y* is one of the spin =1/2 strange hyperons and *B* is an octet baryon, parity violation allows for both *S*- and *P*-wave transitions, and the final states are characterized by the Lee-Yang parameters

**Figure 2.**
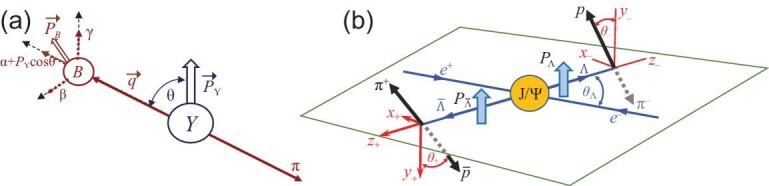
(a) Polarized *Y* → *B*π decay illustrating the α, β, γ dependence of the daughter *B* polarization, where $\vec{q}$ is a vector along the *B* momentum in the *Y* rest frame. (b) The $J/\psi \rightarrow \Lambda \bar{\Lambda }$ reaction. Parity conservation in *J*/ψ decay guarantees that the (cos θ-dependent) Λ and $\bar{\Lambda }$ polarizations are equal and perpendicular to the production plane.

(3) \begin{eqnarray*} \alpha &=& \frac{2{\rm Re}(S^*P)}{|S|^2+|P|^2},\qquad \beta =\frac{2{\rm Im}(S^*P)}{|S|^2+|P|^2}, \nonumber\\ \gamma &=&\frac{|S|^2-|P|^2}{|S|^2+|P|^2}, \end{eqnarray*}

where α^2^ + β^2^ + γ^2^ = 1. If the initial state *Y* has a non-zero polarization $\vec{\mathcal {P}}_Y$, the *B* flight direction in the *Y* rest frame relative to the polarization direction, θ, is distributed as $dN/d\cos \theta \propto 1+\alpha |\vec{\mathcal {P}}_{Y}|\cos \theta$ and, if α is also non-zero, has an explicit parity-violating up-down asymmetry. The polarization of the daughter baryon, $\vec{\mathcal {P}}_B$, depends on ${\mathcal {P}}_Y$, θ and the α, β, γ parameters, as illustrated in Fig. 2(a). If *CP* is conserved, the decay parameters for *Y* and $\bar{Y}$ are equal in magnitude but opposite in sign. (The parameters for $\bar{Y}$ are denoted by $\bar{\alpha }$ and $\bar{\beta }$.) Violations of *CP* symmetry would result in non-zero values for the parameters *A*_*CP*_ and *B*_*CP*_, defined as

(4) \begin{equation*} A_{CP}\equiv \frac{\alpha + \bar{\alpha }}{\alpha - \bar{\alpha }}\quad \text{and}\quad B_{CP}\equiv \frac{\beta + \bar{\beta }}{\beta - \bar{\beta }}. \end{equation*}

Measuring α_Λ_ for Λ → *p*π^−^ decay is not straightforward. Measurements of the up-down parity-violating asymmetry in Λ → *p*π^−^ determine the product $\alpha _{\Lambda }{\mathcal {P}}_{\Lambda }$, where ${\mathcal {P}}_{\Lambda }$ is generally unknown. To extract α_Λ_, the polarization of the final-state proton must be measured. This was done in a series of pre-1975 experiments by scattering the final-state proton on carbon, with a world-average result of α_Λ_ = 0.642 ± 0.013 [92]; this was the PDG value for 43 years, from 1976 until 2019.

BESIII measured α_Λ_ and $\bar{\alpha }_{\Lambda }$ with fully reconstructed $e^+e^-\rightarrow J/\psi \rightarrow (\Lambda \rightarrow p\pi ^-)(\bar{\Lambda }\rightarrow \bar{p}\pi ^+)$ events. For this reaction, the joint angular distribution can be expressed as [93]

(5) \begin{eqnarray*} &&d\Gamma \propto (1+\alpha _{\psi }\cos ^2\theta _\Lambda )\nonumber\\ &&\times [1\!+\!{\mathcal {P}}_{\Lambda }(\cos \theta _{\Lambda })(\alpha _{\Lambda }\cos \theta _- +\bar{\alpha }_{\Lambda }\cos \theta _+)] \nonumber \\ && +\,\alpha _{\Lambda }\bar{\alpha }_{\Lambda }[{\mathcal {F}}_1(\xi )+({1-\alpha _{\psi }^2})^{{1}/{2}}\cos \Delta \Phi {\mathcal {F}}_2(\xi )], \nonumber\\ \end{eqnarray*}

where θ_Λ_ is the Λ production angle relative to the *e*^+^-beam direction (the cos θ_Λ_ distribution is 1 + α_ψ_cos ^2^θ_Λ_); ΔΦ is the complex phase difference between the *A*_+, +_ and *A*_+, −_ helicity amplitudes; and ξ denotes (θ_Λ_, θ_−_, φ_−_θ_+_, φ_+_), where θ_−_, φ_−_ (θ_+_, φ_+_) are the Λ ($\bar{\Lambda }$) decay angles (see Fig. 2(b)). The cos θ_Λ_-dependent Λ (and $\bar{\Lambda }$) polarization is given by

(6) \begin{equation*} {\mathcal {P}}_{\Lambda }(\cos \theta _{\Lambda })=\frac{ ({1-\alpha _{\psi }^2})^{{1}/{2}}\cos \theta _{\Lambda }\sin \theta _{\Lambda }\sin \Delta \Phi }{1+\alpha _{\psi }\cos ^2\theta _{\Lambda }}. \end{equation*}

The Λ polarization is zero if the *A*_+, +_ and *A*_+, −_ helicity amplitudes are relatively real (i.e. ΔΦ = 0), in which case it is apparent from equation (5) that only the product $\alpha _{\Lambda }\bar{\alpha }_{\Lambda }$ can be measured and individual determinations of α_Λ_ and $\bar{\alpha }_{\Lambda }$ cannot be extracted from the data. (Expressions for ${\mathcal {F}}_1(\xi )$ and ${\mathcal {F}}_2(\xi )$ are provided in [93].)

**Figure 3.**
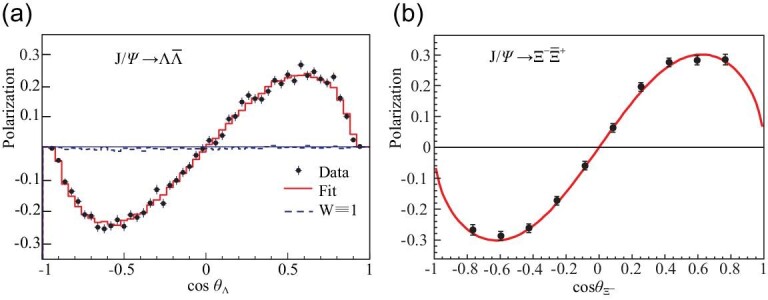
Polarization versus $\cos \theta _{\Lambda (\Xi ^-)}$ for (a) $J/\psi \rightarrow \Lambda \bar{\Lambda }$ [94] and (b) $J/\psi \rightarrow \Xi ^-\bar{\Xi }^+$ [95] events. The red curves are fits to the data; the blue (black) curves are expectations for zero polarization.

When BESIII was being planned, it was generally thought that ${\mathcal {P}}_{\Lambda }\approx 0$ and that $J/\psi \rightarrow \Lambda \bar{\Lambda }$ events would not be useful for *CP* tests. It was somewhat of a surprise when BESIII subsequently discovered that, in fact, the polarization of Λ and $\bar{\Lambda }$ hyperons produced in *J*/ψ decays is substantial [94], as shown in Fig. 3(a). With a sample of 420K fully reconstructed $J/\psi \rightarrow (\Lambda \rightarrow p\pi ^-)(\bar{\Lambda }\rightarrow \bar{p}\pi ^+)$ events in a 1.3B *J*/ψ event sample, BESIII measured $A_{CP}^{\Lambda }=-0.006\pm 0.012\ \pm 0.007$. This null result improved on the precision of the best previous measurement, $A_{CP}^{\Lambda }=+0.013 \pm 0.022$ [87], that was based on 96K $p\bar{p}\rightarrow \Lambda \bar{\Lambda }$ events, by a factor of 2. As a byproduct of this measurement, BESIII made the world’s most precise measurement of α_Λ_ = 0.750 ± 0.010, a result that is more than 5 standard deviations higher than the previous PDG average value. It is likely that all previous measurements were biased by a common systematic problem, probably related to the spin analyzing properties of carbon; the PDG 2019 value for α_Λ_ is solely based on the BESIII value [47].

### Prospects for BESIII *CP* violation studies

The BESIII values for $A_{CP}^{\Lambda }$ and α_Λ_ mentioned in the previous paragraph were realized by an analysis of 1.3B *J*/ψ decays, which is a small subset of BESIII’s total 10B *J*/ψ event sample. The analysis of the full data set is currently underway, which, when completed, will provide a factor-of-3 improvement in sensitivity.

BESIII is currently applying a similar analysis to $J/\psi \rightarrow (\Xi ^-\rightarrow \Lambda \pi ^-)(\bar{\Xi }^+\rightarrow \bar{\Lambda }\pi ^+)$ hyperon pairs, where preliminary results [95] demonstrate that there is substantial transverse Ξ polarization (see Fig. 3(b)). In $\Xi ^-\bar{\Xi }^+$ events, the α_Ξ_ decay parameter influences both the up-down decay asymmetry in the primary Ξ → Λπ process, and the polarization of the daughter Λ hyperons (see Fig. 3(a)) that can be determined from the decay asymmetry in the secondary Λ → *p*π^−^ decay. For a given sample of *J*/ψ decays, the number of fully reconstructed $\Xi ^-\bar{\Xi }^+$ events in which Λ → *p*π^−^ and $\bar{\Lambda }\rightarrow \bar{p}\pi ^+$ are only about one-quarter of the number of reconstructed $J/\psi \rightarrow \Lambda \bar{\Lambda }$ events because of the smaller $J/\psi \rightarrow \Xi ^-\bar{\Xi }^+$ branching fraction and a lower detection efficiency. Nevertheless, this lower event number is compensated by the added information from the daughter Λ decays. As a result, the sensitivity per event for the Ξ^−^ decay parameters is higher than that for Λ parameters with $J/\psi \rightarrow \Lambda \bar{\Lambda }$ events, and simulations show comparable precisions for $\alpha _{\Xi ^-}$ and α_Λ_ [96]. In contrast to Λ → *p*π, where measuring the daughter proton’s polarization is impractical, in Ξ → Λπ decays the daughter Λ polarization is measured and $B_{CP}^{\Xi ^-}$ can be determined; $B_{CP}^{\Xi ^-}$ is potentially more sensitive to new physics than $A_{CP}^{\Xi ^-}$ [97].

In addition to the Λ hyperons produced by $J/\psi \rightarrow \Lambda \bar{\Lambda }$, those produced as daughters in $J/\psi \rightarrow (\Xi ^-\rightarrow \Lambda \pi ^-)(\bar{\Xi }^+\rightarrow \bar{\Lambda }\pi ^+)$ events are also useful for $A_{CP}^{\Lambda }$ measurements. The rms polarization of Λ hyperons produced via $J/\psi \rightarrow \Lambda \bar{\Lambda }$ (see Fig. 3(a)) is $\langle {\mathcal {P}}_{J/\psi ,\Lambda }\rangle _{\rm rms}\approx 0.13$. In contrast, the rms polarization for Λ hyperons produced as a daughter particle in Ξ^−^ → Λπ^−^ decay is $\langle {\mathcal {P}}_{\Xi ^-,\Lambda }\rangle _{\rm rms}\approx |\alpha _{\Xi ^-}|=0.39\pm 0.01$ (see Fig. 2(a)). Thus, $\langle {\mathcal {P}}_{\Xi ^-,\Lambda }\rangle _{\rm rms}\approx 3\langle {\mathcal {P}}_{J/\psi ,\Lambda }\rangle _{\rm rms}$ and, since the $A_{CP}^{\Lambda }$ sensitivity is proportional to $\sqrt{n_{\rm evts}}$ but linear in $\langle {\mathcal {P}}_{\Lambda }\rangle _{\rm rms}$, a Λ from Ξ^−^ → Λπ^−^ decay has 9 times the equivalent statistical power of a Λ from $J/\psi \rightarrow \Lambda \bar{\Lambda }$. Detailed estimates of BESIII’s ultimate statistical error for *A*_*CP*_ with the existing 10B *J*/ψ event sample, including Λ hyperons from Ξ → Λπ decays, are reported in [96] and summarized here in Table 3. The projected ultimate $A_{CP}^{\Lambda }$ sensitivity is ${\mathcal {O}}(2\times 10^{-3})$, which is an order of magnitude improvement on the pre-BESIII result [87].

**Table 3.** The expected numbers of fully reconstructed events and the extrapolated 1σ statistical errors on $\langle \alpha \rangle = (\alpha - \bar{\alpha })/2$ and *A*_*CP*_ from a complete analysis of $J/\psi \rightarrow \Lambda \bar{\Lambda }$, $\Xi ^-\bar{\Xi ^+}$ and $\Xi ^0\bar{\Xi ^0}$ events in BESIII’s 10B *J*/ψ event data sample (from [96]). Here the full reconstruction of the Λ → *p*π^−^ and $\bar{\Lambda }\rightarrow \bar{p}\pi ^+$ decay channels are required.


Reaction	${\mathcal {B}}\ (\times 10^{-4})$	*n*_evts_	δ〈α_Λ_〉	$\delta A_{CP}^{\Lambda }$	$\delta \langle \alpha _{\Xi ^-}\rangle$	$\delta A_{CP}^{\Xi ^-}$	$\delta \langle \alpha _{\Xi ^0}\rangle$	$\delta A_{CP}^{\Xi ^0}$
$J/\psi \rightarrow \Lambda \bar{\Lambda }$	18.9	3200K	0.0010	0.0049				
$J/\psi \rightarrow \Xi ^-\bar{\Xi ^+}$	9.7	810K	0.0018	0.0034	0.0016	0.0039		
$J/\psi \rightarrow \Xi ^0\bar{\Xi ^0}$	11.6	670K	0.0019	0.0041			0.0017	0.0049
Combined			0.0013	0.0023				

## STANDARD MODEL FORBIDDEN PROCESSES

Cross sections for *e*^+^*e*^−^ → *hadrons* in the BESIII accessible *E*_CM_ regions are ${\mathcal {O}}(10~{\rm nb})$ and the experiment typically records ${\mathcal {O}}(10^5)$ events/day. However, at the *J*/ψ resonance peak, the cross section is ≈3.6 μb, and in a typical day of operation BESIII collects ${\mathcal {O}}(10^8)$ events. The cross section at the ψ(2*S*) peak is ≈2 μb and the event rate is ${\mathcal {O}}(5\times 10^7)$ events/day. Thus, at the *J*/ψ and ψ(2*S*) peaks, BESIII has a high rate of events in a very clean experimental environment that is well suited for high sensitivity searches for a number of SM-model forbidden processes. About one-third of the ψ(2*S*) events decay via ψ(2*S*) → π^+^π^−^*J*/ψ, where the triggering on, and detection of only the π^+^π^−^ pair provides an unbiased ‘beam’ of tagged *J*/ψ mesons that can be used to search for decays to final states that would otherwise be undetectable. Table 4 summarizes published BESIII results for forbidden *J*/ψ decay processes.

**Table 4.** Results of the SM forbidden *J*/ψ decay searches performed at BESIII, showing the data sample size, the upper limit at 90% CL on the branching fractions and the best previous results.


Mode	Data	$\mathcal {B}^{\text UL}$ at 90% CL	Ref.	Previous best $\mathcal {B}^{\text UL}$	Ref.
*J*/ψ → γγ	106M ψ(2*S*)	2.7 × 10^−7^	[98]	$\phantom{1}5\times 10^{-6}$	[99]
*J*/ψ → γφ	106M ψ(2*S*)	1.4 × 10^−6^	[98]		
*J*/ψ → *e*μ	225M *J*/ψ	1.6 × 10^−7^	[100]	1.1 × 10^−6^	[101]
$J/\psi \rightarrow \Lambda _c^+e^-$	1.31B *J*/ψ	6.9 × 10^−8^	[102]		

### Search for the Landau–Yang theorem forbidden *J***/ψ → γγ** decay

The Landau–Yang theorem states that a massive spin-1 meson cannot decay to two photons [103,104]. As a consequence, the *J*/ψ → γγ decay mode is strictly forbidden. An unambiguous signal for *J*/ψ → γγ would signal a breakdown of the spin-symmetry theorem of QFT, the underlying framework of the SM and its many proposed new physics extensions. (For a discussion of how QFT might be modified to accommodate a Landau–Yang theorem violation, see [105].)

**Figure 4.**
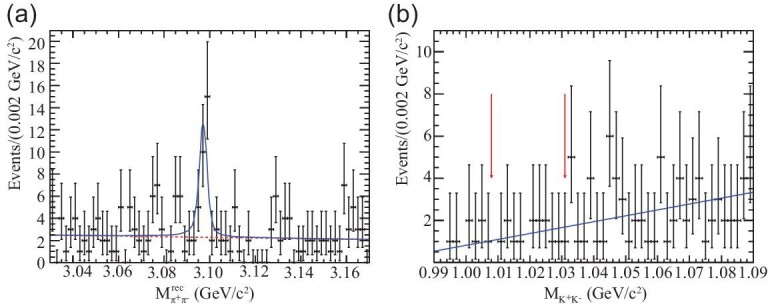
(a) The π^+^π^−^ recoil mass spectrum for selected ψ(2*S*) → π^+^π^−^γγ events. The peak at the π^+^π^−^ recoil mass ≈*m*_*J*/ψ_ = 3.097 GeV is entirely attributable to backgrounds from *J*/ψ → γπ^0^ and γη. (b) The *K*^+^*K*^−^ invariant mass distribution for ψ(2*S*) → γπ^+^π^−^*K*^+^*K*^−^ events with *M*(γ*K*^+^*K*^−^) = *m*_*J*/ψ_ ± 15 MeV. A *J*/ψ → γφ decay would show up as a narrow peak with *M*(*K*^+^*K*^−^) ≈ *m*_φ_ = 1.02 GeV. Both plots are from [98].

The PDG 2018 upper limit, ${\mathcal {B}}(J/\psi \rightarrow \gamma \gamma )<2.7\times 10^{-7}$ [47], is entirely based on a BESIII measurement that uses tagged *J*/ψ mesons that recoil from the π^+^π^−^ system in ψ(2*S*) → π^+^π^−^*J*/ψ decays [98], and is a factor of 20 times more sensitive than previous measurements [99]. In a data sample containing 106M ψ(2*S*) decays, events with two oppositely charged tracks and two γ-rays that satisfy a four-constraint energy-momentum kinematic fit to the π^+^π^−^γγ hypothesis were selected. Figure 4(a) shows the mass recoiling against the π^+^π^−^ tracks where there is a 29 ± 7 event peak at the *J*/ψ mass that is consistent with being entirely due to the expected background from roughly equal numbers of *J*/ψ → γπ^0^ and γη events in which the π^0^ and η decay to a pair of γ-rays with a large energy asymmetry and the low energy γ is undetected either because its energy is below the detection threshold or outside of the fiducial acceptance region of the detector (|cos θ_γ_| > 0.92).

#### Search for the charge-conjugation parity (*C*) violating *J*/ψ → γφ decay

A similar BESIII analysis searched for *J*/ψ → γφ [98]. Although this process does not violate the Landau–Yang theorem, it violates *C* conservation. The weak interactions are known to violate *C* conservation, but the expected branching fractions for weak-interaction-mediated *J*/ψ decays are below the level of 10^−9^ [106]. If *J*/ψ → γφ were seen with a branching fraction that is higher than this, it would imply a violation of *C* conservation in the electromagnetic interaction and be an indicator of new physics. This measurement is based on a search for *J*/ψ decays to γφ; φ → *K*^+^*K*^−^, with tagged *J*/ψ mesons from ψ(2*S*) → π^+^π^−^*J*/ψ decays. In this case kinematically constrained γπ^+^π^−^*K*^+^*K*^−^ events, where the *K*^+^ and *K*^−^ are positively identified as such by the BESIII PID systems and the π^+^π^−^ recoil mass is within ±15 MeV of *m*_*J*/ψ_. Figure 4(b) shows the *K*^+^*K*^−^ invariant mass where there is no sign of a φ → *K*^+^*K*^−^ peak at $M_{K^+K^-}\approx m_{\phi }=1020$ MeV. A 90% CL upper limit on the size of the φ signal is <6.9 events, which translates into a branching fraction upper limit of ${\mathcal {B}}(J/\psi \rightarrow \gamma \phi )<1.4\times 10^{-6}$. This is the first experimental limit for this decay.

### Search for lepton flavor violation in *J***/ψ → *e*μ** decays

The discovery of neutrino oscillations [107] provided clear evidence for violations of lepton flavor conservation (LFV) in the neutrino sector. However, the SM translation of the neutrino results to the charged-lepton sector predicts LFV effects that are proportional to powers of the neutrino masses with branching fractions that are immeasurably small (<10^−51^). Thus, any observation of LFV at levels much higher than this would be clear evidence for new physics, such as grand unified theories or the presence of extra dimensions. Although most attention is given to LFV searches in muon decay, tau decay and μ → *e* conversion experiments, in some theories LFV quarkonium decays, including $V\rightarrow \ell ^-_i\ell ^+_j$ decays, where *i* ≠ *j*, are promising reactions [108]. BESIII searched for the LFV decay *J*/ψ → *e*^−^μ^+^.

**Figure 5.**
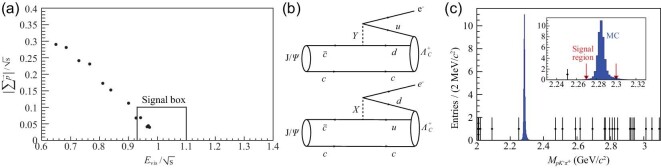
(a) Plot of $|\sum \vec{p}|/\sqrt{s}$ versus $E_{\rm vis}/\sqrt{s}$ for selected *J*/ψ → *e*^−^μ^+^ candidate events in BESIII [100]. (b) Diagrams for leptoquark-mediated $J/\psi \rightarrow e^-\Lambda _{c}^+$ decay as per the model of [109]. (c) The *pK*^−^π^+^ invariant mass distribution for selected, kinematically constrained *J*/ψ → *e*^−^*pK*^−^π^+^ events (from BESIII [102]). The expected shape of a $J/\psi \rightarrow \Lambda _{c}^+e^-$; $\Lambda _{c}^+\rightarrow p K^-\pi ^+$ signal is shown as the blue histogram.

The best previous limit was a 2003 BESII result, ${\mathcal {B}}(J/\psi \rightarrow e^-\mu ^-)<1.1\times 10^{-6}$ [101], that was based on an analysis of a sample of 58M *J*/ψ events. This was improved by a 2013 BESIII result that was based on a sample of 225M *J*/ψ events. In this analysis, the variables $|\sum \vec{p}|/\sqrt{s}$ and $E_{\rm vis}/\sqrt{s}$ are examined for events with two back-to-back and oppositely charged tracks, with one track positively identified as an electron and the other as a muon. Events with detected γ-rays or additional tracks are rejected, and selected events are required to satisfy a four-constraint energy-momentum kinematic fit. The main background is expected to be from *J*/ψ → μ^+^μ^−^ events in which one of the muons passes the electron identification requirements. Figure 5(a) shows a scatterplot of $|\sum \vec{p}|/\sqrt{s}$ versus $E_{\rm vis}/\sqrt{s}$ for selected events, where the four events in the signal box are consistent with the 4.75 ± 1.09 background events that are expected. (This background level corresponds to a muon to electron misidentification probability of ∼10^−7^.) The 90% CL upper limit of ${\mathcal {B}}(J/\psi \rightarrow e^-\mu ^+)<1.6\times 10^{-7}$ that is established [100] is a factor of 7 more stringent than the previous result.

### Search for lepton/baryon number violations in **${\bf J/\psi \rightarrow \Lambda _c^+ e^-}$**

In addition to *CP* violation, another requirement that Sakharov listed for the production of the matter-antimatter symmetry of the universe is the existence of a mechanism for baryon/lepton number violation [5]. Processes that violate baryon ( B) and lepton ( L) number but conserve their difference ( B-L) occur in grand unified theories [109]. Experiments that search for  B-violating decays of the proton have reported lifetime upper limits with spectacular sensitivities: e.g. τ(*p* → *e*^+^π^0^) > 1.6 × 10^34^ years [110]. In contrast, limits for  B-violating decays in the heavy quark sector are sparse and not remotely as sensitive. These include a 90% CL upper limit ${\mathcal {B}}(D^0\rightarrow pe^-)< 1.0\times 10^{-5}$ from CLEO [111] and BaBar branching fraction limits for $B^0\rightarrow \Lambda _{c}^+\ell ^-$ and $B^-\rightarrow \Lambda (\bar{\Lambda })\ell ^-$ (here ℓ = *e*, μ) that range from a few × 10^−6^ for the $\Lambda _{c}^+$ modes to a few × 10^−8^ for the $\Lambda (\bar{\Lambda })$ modes [112].

The only result on  B-violating quarkonium decays is a BESIII upper limit on $J/\psi \rightarrow \Lambda _{c}^+e^-$ that is based on an analysis of a sample of 1.3B *J*/ψ decays. Quark line diagrams for this process in the context of the Pati–Salam model [109] are shown in Fig. 5(b), where  X and  Y are virtual leptoquarks that mediate the decay. BESIII searched for exclusive $J/\psi \rightarrow \Lambda _{c}^+e^-$ decay events where the $\Lambda _{c}^+$ decays to *pK*^−^π^+^ (${\mathcal {B}}=6.3\%$). The *pK*^−^π^+^ invariant mass distribution for candidate events, shown as data points in Fig. 5(c), has no events in the mass interval that is ±4 times the resolution and centered on the $\Lambda _{c}^+$ mass. The absence of any event candidates translates into a 90% CL frequentist upper limit of ${\mathcal {B}}(J/\psi \rightarrow \Lambda _{c}^+e^-)<6.9\times 10^{-8}$ [102].

## SEARCHES FOR NEW, BEYOND THE STANDARD MODEL PARTICLES

In spite of the success of the SM, particle physics still faces a number of mysteries and challenges, including the origin of elementary particle masses and the nature of dark matter (DM). The Higgs mechanism [113] is a theoretically attractive way to explain the mass of elementary particles. However, the SM relation for the Higgs mass is a potentially divergent infinite sum of quadratically increasing terms that somehow add up to the finite value *m*_Higgs_ = 125 GeV, a SM feature that many theoretical physicists consider to be *unnatural* [114]. The existence of DM is inferred from a number of astrophysical and cosmological observations [115]. One possibility is that DM may be comprised of electrically neutral, weakly interacting, stable particles with a mass at the electroweak scale. However, none of the SM particles are good DM candidates and, from the perspective of theory and phenomenology, this implies that the SM is deficient and the quest for a more fundamental theory beyond the SM is strongly motivated. In some extensions of the SM, the naturalness and DM problems can be solved at once.

The naturalness problem can be solved by supersymmetry (SUSY) [116], where every SM particle has an as yet undiscovered partner with the same quantum numbers and gauge interactions but differs in spin by $\frac{1}{2}$. The most economical and intensively studied version of SUSY is the minimal supersymmetric model (MSSM) [116], with superpartners that include

$$\begin{array}{lll} \text{spin zero} & sfermions:\; & \text{left handed \textbackslash{}tilde\{f\}\_L, right}\\ & & \text{handed \textbackslash{}tilde\{f\}\_R},\\ \text{spin-\textbackslash{}frac\{1\}\{2\}} & gauginos: & \text{a bino \textbackslash{}tilde\{B\}, three winos \textbackslash{}tilde\{W\}\_i,}\\ & & \text{gluinos \textbackslash{}tilde\{g\}},\\ \text{spin-\textbackslash{}frac\{1\}\{2\}} & higgsinos: & \text{two \textbackslash{}tilde\{H\}\_i}. \end{array}$$

The two higgsinos can mix with the bino and the three winos to produce two *chargino*$\chi _{1,2}^\pm$ and four *neutralino*$\chi _{1,2,3,4}^0$ physical states. A discrete symmetry called *R*-parity is introduced to make the lightest SUSY particle, usually the $\chi _1^0$, stable, which makes it a nearly ideal DM candidate that is often denoted as simply χ. A further extension is the so-called next-to-minimal MSSM (NMSSM) [117–119], in which a complex isosinglet field is added. The NMSSM has a rich Higgs sector containing three *CP*-even, two *CP*-odd, and two charged Higgs bosons. The mass of the lightest *CP*-odd scalar Higgs boson, the *A*^0^, may be less than twice the mass of charm quark, in which case it would be accessible at BESIII.

Although the lightest neutralino is an attractive DM candidate, the lack of any experimental evidence for it in either LHC experiments or direct detection experiments suggests that DM might be more complex than the neutralino of the SUSY models. Attempts to devise a unified explanation have led to a vast and diverse array of dark-sector models. These models necessarily have several sectors: a *visible sector* that includes all of the SM particles, a *dark sector* of particles that do not interact with the known strong, weak or electromagnetic forces and a *portal sector* that consists of particles that couple the visible and dark sectors. The latter may be vectors, axions, Higgs-like scalars or neutrino-like fermions [120,121], of which vectors are the most frequently studied. The simplest scenario for the vector portal invokes a new force that is mediated by a *U*(1) gauge boson [122] that couples very weakly to charged particles via kinetic mixing with the SM photon γ, with a mixing strength ϵ that is in the range between 10^−5^ and 10^−2^ [123]. This new boson is variously called a dark photon, hidden photon or *U* boson, and is denoted as γ^′^. The γ^′^ mass is expected to be low, of the order of MeV/*c*^2^ to GeV/*c*^2^ [123] and, thus, it could be produced at the BEPCII collider in a variety of processes, depending on its mass.

### Search for *A*^0^, **γ^′^** and invisible decays of light mesons

Both the light *CP*-odd NMSSM Higgs boson *A*^0^ and dark photon γ^′^ have been searched for by BESIII. Since it is Higgs-like, the *A*^0^ couples to SM fermions with a strength proportional to the fermion mass. For an *A*^0^ with a mass below the τ pair production threshold, the decay *A*^0^ → μ^+^μ^−^ is expected to be dominant. The *A*^0^ can also serve as a portal to the dark sector with the invisible-final-state decay process $A^0\rightarrow \chi \bar{\chi }$. Similarly, as a portal between the SM and dark sectors, the γ^′^ can, in turn, either decay to $\chi \bar{\chi }$, or visibly to a pair of light leptons or quarks, provided it is kinematically allowed.

**Figure 6.**
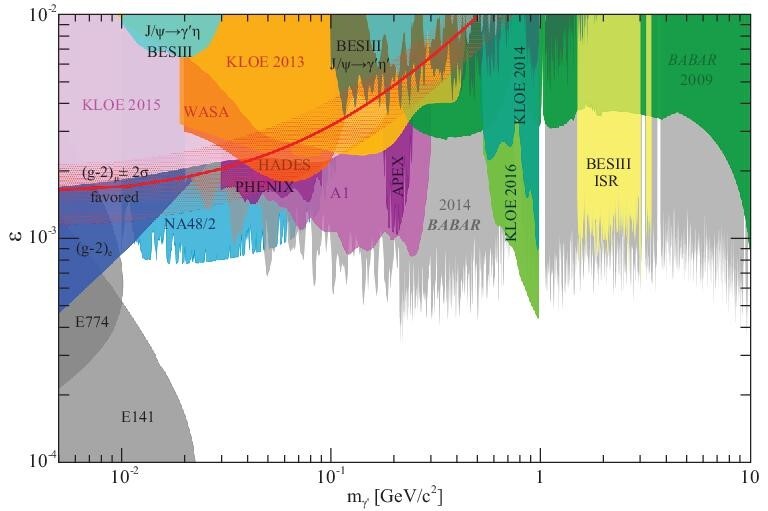
Exclusion limits at the 90% confidence level for the mixing strength parameter ϵ as a function of the dark photon mass $m_{\gamma ^{\prime }}$. Also shown are exclusion limits from other experiments. The ϵ values that would explain the discrepancy between the measured and SM-calculated value of the anomalous magnetic moment of the muon [134] are displayed as the bold solid red line along with its 2σ band. Plot is from [129], overlaid with the BESIII limits of *J*/ψ → ηγ^′^ and *J*/ψ → η^′^γ^′^.

BESIII results on searches for the *A*^0^, γ^′^ and invisible decays of light meson states are summarized in Table 5. The *A*^0^ was searched for in *J*/ψ → γ*A*^0^ (*A*^0^ → μ^+^μ^−^) and ψ(2*S*) → π^+^π^−^*J*/ψ (*J*/ψ → γ*A*^0^) (*A*^0^ → μ^+^μ^−^) decay candidate events in BESIII’s *J*/ψ [124] and ψ(2*S*) [125] data samples. The sensitivity obtained with the *J*/ψ data is 5 times better than that with the ψ(2*S*) data. The combination of BaBar [126] and BESIII [124] measurements constrain the *A*^0^ to be mostly singlet. BESIII published three results on dark photon (γ^′^) searches in *J*/ψ and ψ(3770) decays with resulting 90% CL exclusion regions for ϵ as a function of the dark photon mass that are shown in Fig. 6. BESIII dark photon searches in *J*/ψ → ηγ^′^ (γ^′^ → *e*^+^*e*^−^) decays [127] and *J*/ψ → η^′^γ^′^ (γ^′^ → *e*^+^*e*^−^) decays [128] were among the first searches that were based on these channels [129]. BESIII results for dark photon searches in *e*^+^*e*^−^ → γ_ISR_γ^′^(γ^′^ → ℓ^+^ℓ^−^, ℓ = *e*, μ) initial state radiation events were based on 2 years of data taking and are competitive with BaBar results [130] based on 9 years of running. Invisible decays of light mesons that are produced in *J*/ψ decays were also searched for at BESIII. These include the first measurements for the ω and φ vector mesons that are copiously produced via *J*/ψ → ωη and φη decays [131]. For *J*/ψ → φη ($\eta \rightarrow \emph {invisible}$) and *J*/ψ → φη^′^ ($\eta ^{\prime }\rightarrow \emph {invisible}$) decays, the BESIII limits [132] are factors of 6 and 3 improvements over previous results from BESII [133]. These results provide complementary information to studies of the nature of DM and constrain parameters of the phenomenological models.

**Table 5.** BESIII results on searches for the light *CP*-odd Higgs boson *A*^0^, the dark photon γ^′^, and invisible decays of quarkonium and light mesons. The first column lists the decay modes and the third column lists the measured 90% CL branching fractions upper limits. For the visible dark photon decays, the corresponding γ − γ^′^ mixing strength ϵ limits are shown in the fourth column.


Mode	Data	$\mathcal {B}^{\text UL}$ at 90% CL	ϵ ( × 10^−3^)	Ref.
*J*/ψ → γ*A*^0^( → μ^+^μ^−^)	225M *J*/ψ	(2.8 − 495.3) × 10^−8^		[124]
ψ^′^ → ππ*J*/ψ( → γ*A*^0^( → μ^+^μ^−^))	106M ψ(2*S*)	(4 − 210) × 10^−7^		[125]
*J*/ψ → ηγ^′^( → *e*^+^*e*^−^)	1.31B *J*/ψ	(1.9 − 91.1) × 10^−8^	10 − 1	[127]
*J*/ψ → η^′^γ^′^( → *e*^+^*e*^−^)		(1.8 − 20) × 10^−8^	3.4 − 26	[128]
*e* ^+^ *e* ^−^ → γ_ISR_γ^′^( → *e*^+^*e*^−^/μ^+^μ^−^)	2.93 fb^−1^ ψ(3770)		0.1 − 1	[129]
$J/\psi \rightarrow \eta \omega (\omega \rightarrow \emph {invisible})$	1.31B *J*/ψ	7.3 × 10^−5^		[131]
$J/\psi \rightarrow \eta \phi (\phi \rightarrow \emph {invisible})$		1.7 × 10^−4^		
$J/\psi \rightarrow \phi \eta (\eta \rightarrow \emph {invisible})$	225M *J*/ψ	1.0 × 10^−4^		[132]
$J/\psi \rightarrow \phi \eta ^{\prime }(\eta ^{\prime }\rightarrow \emph {invisible})$		5.3 × 10^−4^		

## INTERACTIONS WITH OTHER EXPERIMENTS

The standard model of particle physics is a seamless structure in which measurements in one sector have profound impact on other, seemingly unrelated areas. Thus, for example, BESIII measurements of strong-interaction phases in hadronic decays of charmed mesons provide important input into determinations of the *CP*-violating angle γ in *B*-meson decays by BelleII and LHCb. Similarly, BESIII measurements of the annihilation cross section for *e*^+^*e*^−^ → *hadrons* at energies below 2 GeV provide critical input to the interpretation of high-energy tests of the SM at the Higgs (126 GeV) and top-quark (173 GeV) mass scales as well as the measurements of (*g* − 2)_μ_, the anomalous magnetic moment of the muon. The relation between BESIII measurements of strong phases in the charmed sector to *CP*-violating measurements in the beauty sector are discussed elsewhere in this journal volume [53]. Here we briefly review the impact of the BESIIII cross-section results on the interpretation of (*g* − 2)_μ_ measurements.

### BESIII impact on the determination of (*g* − 2)**_μ_**

**Figure 7.**
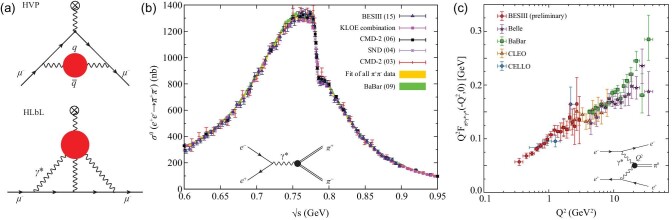
(a) Hadron vacuum polarization (HVP) and hadron light-by-light scattering (HLbL) contributions to the SM calculation of (*g* − 2)_μ_. The red circles represent hadronic contributions. (b) Measurements of σ(*e*^+^*e*^−^ → π^+^π^−^) from SND [140], CMD-2 [141,142], BaBar [143], KLOE [144] and BESIII [145]. The structure near *E*_CM_ = 0.78 GeV is caused by interference between ρ → π^+^π^−^ and ω → π^+^π^−^ (from [146]). (c) Preliminary BESIII results for the π^0^ form factor [147] together with results from CELLO [148], CLEO [149], BaBar [150] and Belle[151] (from [136]).

The measured value of (*g* − 2)_μ_ from BNL experiment E821 [135] is ∼3.7 standard deviations higher than the SM prediction [136], a discrepancy that has inspired elaborate follow-up experiments at Fermilab [137] and J-PARC [138]. As illustrated in Fig. 7(a), the SM predicted value for (*g* − 2)_μ_ is very sensitive to the effects of hadronic vacuum polarization (HVP) of the virtual photon, which are about 100 times larger than the current experimental uncertainty. The contributions from higher-order radiative corrections to the μ-γ vertex, so-called hadron light-by-light (HLbL) scattering, is of the same order as the current experimental error, but it has a 20% theoretical uncertainty that will be comparable to the expected error from the new round of experiments.

Vacuum polarization also has critical influence on precision tests of the electroweak theory, which rely on a precise knowledge of α(*s*), the running QED coupling constant. Because of vacuum polarization, $\alpha ^{-1}(m^2_Z)=128.95\pm 0.01$ [139], about 6% below its long distance value of α^−1^(*s* = 0) = 137.04. About half of this change is due to HVP.

#### Precision measurement of vacuum polarization of virtual photons

Since HVP effects are non-perturbative, they cannot be directly computed from first-principle QCD. Recent computer-based lattice QCD (LQCD) calculations have made significant progress but the uncertainties are still large [152,153]. The most reliable determinations to date of HVP contributions to (*g* − 2)_μ_ and $\alpha (m^2_Z)$ use dispersion relations with input from experimental measurements of cross sections for *e*^+^*e*^−^ annihilation into hadrons [136]. The data used for the most recent determinations are mostly from the SND [140], BaBar [143], BESIII [145], CMD-2 [141,142] and KLOE [144] experiments. BaBar and KLOE operations have been terminated, leaving SND, CMD-3 [154] and BESIII as the only running facilities with the capability to provide the improvements in precision that will be essential for the evaluation of (*g* − 2)_μ_ with a precision that will match those of the new experimental measurements.

With data taken at *E*_CM_ = 3.773 GeV (primarily for studies of *D*-meson decays), BESIII measured the cross sections for *e*^+^*e*^−^ → π^+^π^−^ at *E*_CM_ between 0.6 and 0.9 GeV [145], which covers the ρ → π^+^π^−^ peak, the major contributor to the HVP dispersion relation integral. These measurements used initial state radiation (ISR) events in which one of the incoming beam particles radiates a γ-ray with energy *E*_ISR_ = *xE*_CM_/2 before annihilating at a reduced CM energy of $E_{\rm CM}=\sqrt{1-x}E_{\rm CM}$. The relative uncertainty of the BESIII measurements is 0.9%, which is similar to the precisions of the BaBar [143] and KLOE [144] results. The BESIII measured values agree well with KLOE results for energies below 0.8 GeV, but are systematically higher at higher energies; in contrast, BESIII results agree with BaBar at higher energies, but are lower at lower energies. Detailed comparisons are shown in Fig. 7(b). Nevertheless, the contributions of *e*^+^*e*^−^ → π^+^π^−^ to the (*g* − 2)_μ_ HVP calculation from these experiments have overall agreement within 2 standard deviations, and the observed ∼3.7 standard deviation difference between the calculated muon magnetic moment value and the E821 experimental measurement persists.

#### Experimental input for data-driven HLbL determinations

The HLbL scattering contribution to the SM (*g* − 2)_μ_ value has a hadron loop (see Fig. 7(a)) that is non-perturbative and in a more complex environment than the HVP loop. As a result, its determination is not straightforward and has a rather volatile history (see [155]). In this case, the loop integral is dominated by single mesons (π^0^, η, η^′^) but, since they couple to virtual photons, their time-like form factors at low *Q*^2^ values are involved. Until now, only high *Q*^2^ measurements of these form factors have been reported and models were used to extrapolate these to the low *Q*^2^ regions of interest. Recently, however, BESIII reported preliminary π^0^ form-factor results for *Q*^2^ values in the range 0.3–1.5 GeV^2^ [147] (see Fig. 7(c)). These are the first experimental results that include momentum transfers below *Q*^2^ = 0.5 GeV^2^, the relevant region for HLbL calculations. These, and measurements of the η and η^′^ form factors that are currently underway, will reduce the model dependence and, thus, the theoretical errors of the HLbL contribution to (*g* − 2)_μ_.

### Prospects for (*g* − 2)**_μ_**-related measurements at BESIII

Currently, the precision of the (*g* − 2)_μ_ measurement (54 ppm [135]) is comparable to that of the SM calculation (37 ppm [136]). However, since a 4-fold improvement in the experimental precision is imminent, improvements in the theoretical precision are needed. These will require improved experimental input for the data-driven evaluations of the HVP and HLbL terms and/or improved LQCD calculations. BESIII is improving the σ(*e*^+^*e*^−^ → *hadrons*) measurements used for the HVP term and providing light meson form factors for the HLbL determination. Moreover, precision BESIII measurements of various decay constants and form factors provide calibration points that are used to validate LQCD techniques.

## SUMMARY AND PERSPECTIVES

In the search for new, beyond the standard model physics, there is no compelling theoretical guidance for where it might first show up. It may first appear at the energy frontier that is explored at the LHC, or at the intensity frontier that is pursued at lower energies. (Interestingly, the current most prominent candidate for BSM physics is the ∼3.7σ discrepancy in (*g* − 2)_μ_, which is about as far removed from the energy frontier as one can get.) A key aspect of any experiment is *reach*, i.e. the range of unexplored SM-parameter space that is explored. In this quest, BESIII is accumulating huge numbers of *J*/ψ and ψ(2*S*) events that support high sensitivity searches for low-mass non-SM particles, SM-forbidden decay processes and non-SM *CP* violations in hyperon decays. In addition, high statistics samples of *D* and *D*_*s*_ mesons produced just above threshold in very clean experimental environments provide the means to search for new physics in the (*u*, *d*)-(*c*, *s*) quark sector with the world’s best precision. BESIII is continuing the BES program’s long history of steadily improving the precision of *e*^+^*e*^−^ → *hadrons* annihilation cross-section measurements and light meson form-factor determinations that are used to evaluate HVP and HLbL corrections that are needed for the interpretation of SM tests being done by other experiments.

Results highlighted here are primarily based on data samples that were accumulated at the peaks of the narrow *J*/ψ and ψ(2*S*) charmonium states and the $\psi (3770)\rightarrow D\bar{D}$ resonance. These data samples correspond to 1.3B *J*/ψ events, 448M ψ(2*S*) events and a 2.93 fb^−1^ integrated luminosity exposure at ψ(3770). Thanks to the excellent operation of the BEPCII collider, BESIII recently collected a total of 10B *J*/ψ events that are now being analyzed. And, as this report is being written, a data-taking run is in progress that has the goal of collecting a total of 4M ψ(2*S*) events. When this run is completed, the BEPCII energy will be set at the ψ(3770) peak, where it will stay until the total exposure at this energy reaches 20 fb^−1^. These nearly 10-fold increases in the amount of available data will extend the BESIII discovery reach for new, BSM physics by a factor of 3 for most channels, and by almost an order of magnitude for processes with zero backgrounds.
